# Editorial: Integrative analysis of single-cell and/or bulk multi-omics sequencing data

**DOI:** 10.3389/fgene.2022.1121999

**Published:** 2023-01-04

**Authors:** Geng Chen, Rongshan Yu, Xingdong Chen

**Affiliations:** ^1^ Stemirna Therapeutics Co., Ltd., Shanghai, China; ^2^ Department of Computer Science, School of Informatics, Xiamen University, Xiamen, China; ^3^ State Key Laboratory of Genetic Engineering, Human Phenome Institute, School of Life Sciences, Fudan University, Shanghai, China

**Keywords:** multi-omics, single-cell sequencing, bulk sequencing, integrative analysis, data integration

## Introduction

Each type of omics data including genomics, epigenomics, transcriptomics, proteomics, metabolomics, and metagenomics mainly provides the profile of one particular layer for a cell or sample (Hasin et al., 2017). Integrative analysis of multi-omics data could enable a more comprehensive dissection from different perspectives, which may facilitate a better and deeper understanding of the underlying molecular functions and mechanisms (Li et al., 2021). With the innovation and development of sequencing technologies, various single-cell and bulk profiling technologies have been developed and applied to a diversity of biological and clinical research (Lei et al., 2021; Li et al., 2021; Jiang et al., 2022). Bulk sequencing approaches allow the elucidation of each sample at the cell-population level, providing the averaged profile of a multitude of cells. By contrast, single-cell sequencing methods can interrogate thousands of cells at single-cell resolution for a given sample simultaneously. Joint analysis of multi-omics data generated from bulk and single-cell sequencing protocols could effectively facilitate the translation of basic science to practical applications (Stuart and Satija, 2019; Leng et al., 2022). On the other hand, the sample/cell scale and data size are growing rapidly in biomedical investigation. Thus, novel bioinformatics approaches are also in urgent need to more efficiently and robustly integrate distinct types of omics data.

**FIGURE 1:**
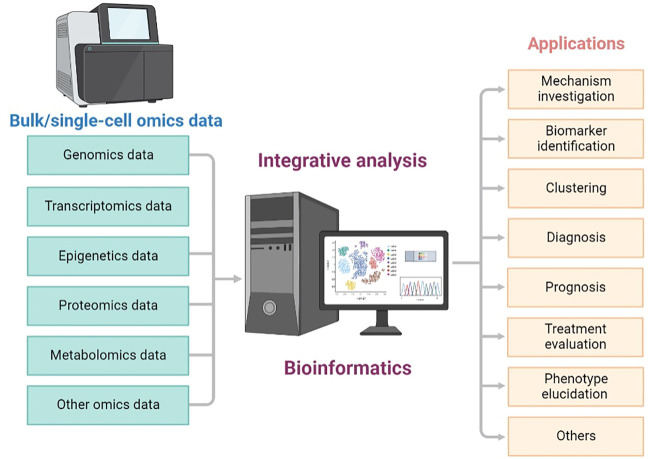
Overview of integrative analysis of multi-omics data generated from different bulk and single-cell sequencing technologies.

Since multi-omics strategies could be more powerful than single omics, combining different types of single-cell or bulk sequencing data for a more comprehensive exploration has become increasingly popular and important (Figure 1). In this Research Topic on Integrative Analysis of Single-Cell and/or Bulk Multi-omics Sequencing Data, we planned to collect novel findings and methods related to analyzing bulk and single-cell multi-omics or multimodal data with a systematic strategy. In total, 12 original research articles and one case report were published in this Research Topic, covering multi-omics-based cancer dissection, comparison of different data integration methods, and database construction for expression examination in various tissues. Here we concisely summarize and discuss the main results revealed in these studies.

## Studies published in this research topic

Guo et al. found that the mutations in TP53 and KRAS were significantly associated with the poor prognosis of intrahepatic cholangiocarcinoma (ICC). They further classified the ICC patients into different subgroups based on the mutation feature of TP53 and KRAS, which could benefit the clinical management of ICC. Johann et al. uncovered that the mutations of AKT1 and TP53 signaling pathways were closely associated with the pulmonary sclerosing pneumocytoma (PSP) through integrative analysis of genomic, transcriptomic, radiomic, and pathomic data. The insights into the underlying etiology and molecular behavior of PSP gained in this study may benefit corresponding therapy. Gao et al. constructed an effective prognostic model for breast cancer using the differentially expressed genes among distinct glycosylation patterns. Their results highlight the value and importance of risk score characterization based on glycosylation patterns for predicting the overall survival and immune infiltration of breast cancer patients. Hao et al. identified two subgroups of MYC signaling inhibition and activation for lung adenocarcinoma (LUAD) through joint analysis of genomics, transcriptomics, and single-cell sequencing data from multiple cohorts. The two LUAD subgroups discovered by them exhibited significant differences in terms of prognosis, genomic variations, immune microenvironment, as well as clinical features. Additionally, Jiang et al. built and validated a model for predicting the prognosis of LUAD by integrating bulk and single-cell RNA-seq data. They also detected two distinct subtypes of LUAD patients that differed in prognosis and immune characteristics. Sun et al. systematically analyzed the transcriptome of synovial sarcoma in terms of gene expression, alternative splicing, gene fusion, and circular RNAs. Their integrative analysis provided new insights into the transcriptomic profile and the underlying molecular mechanism of synovial sarcoma. Wang et al. constructed a clinical diagnostic map and a cluster prediction model for glioblastoma based on the methylation, expression, and single-cell sequencing data. The classification method developed by them could potentially promote the analysis of methylation heterogeneity for the promoter CpG islands in glioblastoma. Zhao et al. revealed high cellular heterogeneity in both malignant and immune cells of diffuse large B-cell lymphoma (DLBCL). They provided novel insights into the transcriptional dynamics of the tumor microenvironment for DLBCL. Zhang et al. established a prognostic model based on eight genes (DEFB1, AICDA, TYK2, CCR7, SCARB1, ULBP2, STC2, and LGR5) for predicting the overall survival of head and neck squamous cell carcinoma (HNSCC) patients. The low-risk and high-risk groups of HNSCC separately showed higher and lower immune scores, thus those eight gene signatures have the potential to be used in the clinical management of HNSCC. Li et al. classified hepatocellular carcinoma patients into high-necroptosis and low-necroptosis groups, which had a significant difference in survival time. They found that the high-necroptosis patients were with an enriched expression of immune checkpoint-related genes and could benefit from certain immunotherapy. Wang et al. uncovered four dysregulated oncogenic signaling pathways and identified related potential prognostic biomarkers for pan-cancer through systematic analysis of the TCGA multi-omics data. Their results could facilitate a better understanding of the function of oncogenic signaling pathways in human pan-cancer. Kujawa et al. systematically evaluated the influence of six different data integration methods on single-cell analysis. They found that ComBat-seq (Zhang et al., 2020), limma (Leek et al., 2012), and MNN (Haghverdi et al., 2018) could effectively reduce batch effects and preserve the differences between distinct biological conditions. Deng et al. constructed a gene expression omnibus database named ECO (https://heomics.shinyapps.io/ecodb/) for mouse endothelial cells based on the sequencing data of 203 samples from 71 different conditions. ECO could enable researchers to friendly explore endothelial expression profiles of diverse tissues in conditions of certain genetic modifications, disease models, and other stimulations *in vivo*.

## Summary and perspectives

The studies published on this Research Topic discovered meaningful results and offered new insights into corresponding biomedical research. As we all know that the cost of sequencing technologies is gradually decreasing, which can facilitate the conduction of multi-omics investigations. Bulk and single-cell protocols have their own advantages and limitations. Compared to single-cell sequencing methods, bulk approaches do not need living cells and the experimental procedures are usually simpler (Li et al., 2021). Dissecting large-scale samples is more affordable for bulk strategies, but bulk data can not effectively provide cellular heterogeneity information. Single-cell sequencing allows a better understanding of cell-to-cell variations and molecular dynamics at single-cell resolution. However, existing single-cell technologies for generating different types of omics data still suffer lower capture efficiency and higher technical noise compared to traditional bulk protocols (Mustachio and Roszik, 2022; Wen and Tang, 2022). Therefore, bulk and single-cell approaches are complementary, the combination of bulk and single-cell data is valuable for getting both cell-population and single-cell level perspectives (Li et al., 2021). For example, the proportion of cell subtypes for large-scale bulk data could be deconvoluted with the cell-type-specific signatures inferred from the single-cell data of a small number of samples (Aibar et al., 2017; Wang et al., 2019; Zaitsev et al., 2019; Decamps et al., 2020; Lin et al., 2022). The biomarkers identified in single-cell sequencing data can be further correlated to the outcomes of patients to assess their potential clinical value using corresponding bulk data from public databases such as The Cancer Genome Atlas (TCGA) (Weinstein et al., 2013).

Collectively, joint analysis of bulk and single-cell multi-omics data can help us gain a more comprehensive and systematic view of biological and clinical samples. The innovation of various omics profiling technologies and related machine learning methods for integrating different types of data will further make multi-omics exploration more feasible and easier. We hope the studies published on this Research Topic will inspire related biomedical researchers to better understand the benefit and value of multi-omics strategies.
